# An Unusual Endoscopic Image of a Submucosal Angiodysplasia

**DOI:** 10.1155/2012/186065

**Published:** 2012-09-19

**Authors:** Rita Carvalho, Nuno Almeida, Manuela Ferreira, Pedro Amaro, António Bernardes, Maria Augusta Cipriano, J. M. Romãozinho, Hermano Gouveia, Carlos Sofia

**Affiliations:** ^1^Gastroenterology Department, Coimbra University Hospital, 3000-075 Coimbra, Portugal; ^2^General Surgery Department, Coimbra University Hospital, 3000-075 Coimbra, Portugal; ^3^Pathology Department, Coimbra University Hospital, 3000-075 Coimbra, Portugal

## Abstract

Obscure gastrointestinal bleeding is responsible for 2–10% of the cases of digestive bleeding. Angiodysplasia is the most common cause. The authors report a case of a 70-year-old female patient admitted to our Gastrointestinal Intensive Care Unit with a significant digestive bleeding. Standard upper and lower endoscopy showed no abnormalities, and we decided to perform a capsule enteroscopy that revealed a submucosal nodule with active bleeding in the jejunum. An intraoperative enteroscopy confirmed the presence of a small submucosal lesion with a central ulceration, and subsequently a segmental enterectomy was performed. Surprisingly, the histopathological diagnosis was angiodysplasia. The patient remains well after a two-year period of follow-up. We present this case of obscure/overt gastrointestinal bleeding to emphasize the role of capsule and intraoperative enteroscopy in the evaluation of these situations, and because of the unusual endoscopic appearance of the angiodysplasia responsible for the hemorrhage.

## 1. Case Presentation

Obscure gastrointestinal bleeding may present as obscure or overt (OOGIB). The overt form is sometimes massive but the new enteroscopy methods, namely, capsule endoscopy (CE) and deep enteroscopy, revolutionized the approach to this kind of gastrointestinal bleeding. Even so, intraoperative enteroscopy (IOE) is still an important diagnostic/therapeutic examination that must be considered in these patients. 

The authors report a case of a 70-year-old female patient that was admitted to our Gastrointestinal Intensive Care Unit, transferred from another hospital, with the diagnosis of OOGIB, presenting with persistent hematochezia. The patient had history of mitral valve prosthesis and was on warfarin therapy.

On admission she was hemodynamically stable despite maintained hematochezia. Her physical examination was otherwise normal.

Laboratory investigations showed anemia (hemoglobin of 7,9 g/dL, medium globular volume of 70 fL) and an international normalized ratio of 1,37. 

An upper gastrointestinal endoscopy and a total colonoscopy had already been performed in the hospital of origin. Even so, we decided to repeat standard endoscopic examinations, but no bleeding lesions were found.

Although the patient remained hemodynamically stable, the hemorrhage persisted, with relevant transfusion requirements (1-2 units of red blood cells/24 hour period). 

**Figure 1:**
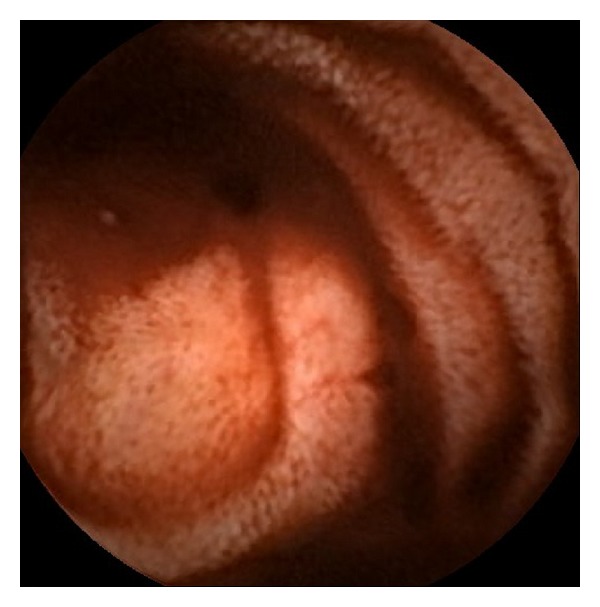
Image of capsule enteroscopy showing a submucosal nodule of small size, with active bleeding, in the jejunum.

At day four of hospitalization, we decided to perform a CE. A submucosal nodule of small size, with active bleeding, was identified in the jejunum (Figure 1). Although there was blood from this point on, no other sources of bleeding were seen. 

**Figure 2:**
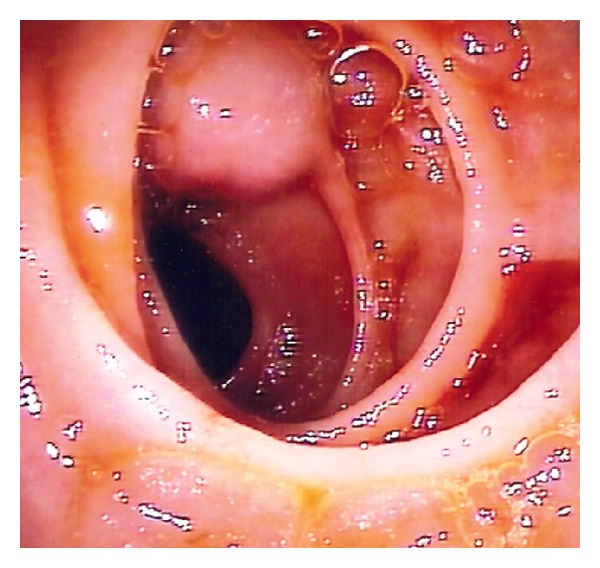
Image of the intraoperative enteroscopy where is visible a small submucosal lesion with central ulceration.

**Figure 3:**
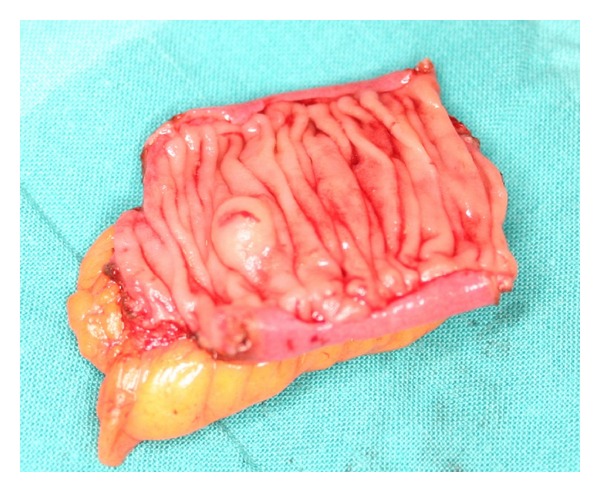
Image of the resected segment of jejunum with a small nodular lesion.

This lesion was suggestive of a bleeding gastrointestinal stromal tumor (GIST), so the decision was to advance to surgery with per-oral IOE. At 40 centimeters from the ligament of Treitz, a small submucosal tumor with central ulceration was identified (Figure 2). At the time of the surgery there was no active bleeding, but there was fresh blood in the lumen. Subsequently, a segmental enterectomy with removal of the lesion was performed (Figure 3). 

**Figure 4:**
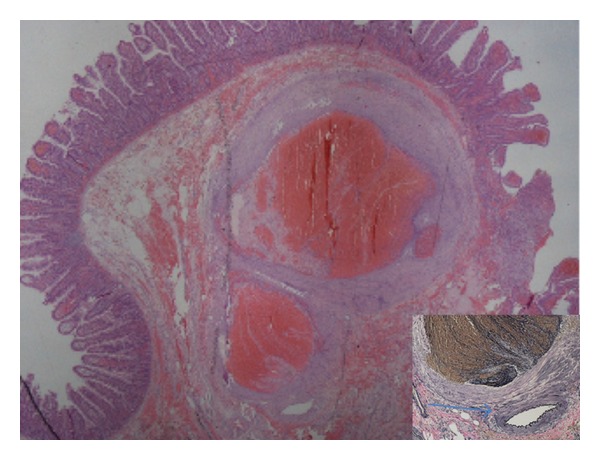
HE 20X revealing a dilated submucosal vein containing organized thrombi. In the right lower quadrant, an image of Verhoeff 100X showing the venous proliferation next to the artery (arrow).

The macroscopic analysis revealed a segment of small bowel of 3,5 centimeters with a polypoid lesion of 0,7 centimeters. In microscopic analysis the protruded lesion was a dilated submucosal vein that bulged into the lumen, with partial venous thrombosis (Figure 4). The histopathological diagnosis was angiodysplasia. The patient was discharged on the 15th postoperative day, and remains well after a two-year follow-up period.

## 2. Discussion

Obscure bleeding is defined as hemorrhage from the gastrointestinal (GI) tract that persists or recurs without an obvious etiology after upper endoscopy, colonoscopy, and radiological evaluation, and is subdivided into overt or occult, depending upon the presence or absence of clinically evident hemorrhage [1]. It is responsible for 2–10% of the cases of digestive bleeding [2]. It is considered severe (1% of all cases) if any of the following is present: overt presentation, recurrent episodes of acute bleeding, transfusion dependence, and need for hospitalization [3].

There are several possible causes of obscure GI bleeding. Their relative frequency is not well defined but probably depends upon age, with older patients having more likely bleeding from vascular lesions. 

Angiodysplasias are the most common vascular lesions encountered in the GI tract and account for 30–40% of GI bleeding [4]. They are composed of ectatic, dilated, thin-walled vessels that are lined by endothelium alone or by smooth muscle. Histological examination demonstrates dilated vessels in the mucosa and submucosa. The pathogenesis is not well understood but can be congenital, secondary to chronic venous obstruction, to chronic mucosal ischemia, or to local ischemia related to cardiac, pulmonary, or vascular pathologies [5]. Endoscopically the most common appearance is a flat red lesion. They occur most often in the ascending colon and cecum, but in 10% of cases can appear only in the small bowel. They may be multiple and coexist in different gastrointestinal locations.

The diagnosis of those located in small bowel is not always easy. IOE has been considered the gold standard with a high diagnostic yield, but nowadays it is reserved for patients in whom other methods for visualizing the small bowel have not been successful, or for patients who need surgery. 

CE allows the examination of the entire small bowel with similar diagnostic yield, and double-balloon enteroscopy permits real-time exploration with the advantage of therapeutic approach [2]. 

In our institution and in this particular clinical case we used early CE because it has no major complications, it is easy to perform with no discomfort to the patient [2], and we had the possibility to read it right after the examination. This strategy has the advantage of determining the next step. If an endoscopic treatment is feasible, CE helps to determine the route of insertion of deep enteroscopy; if a tumor is detected, we can opt for direct surgical approach.

There are several endoscopic modalities of treatment such as sclerotherapy, band ligation, and specially argon plasma coagulation that can be used with high effectiveness. Surgical resection can be a definitive treatment in lesions clearly identified as the source of hemorrhage, as in our clinical case, but bleeding may recur from other lesions in gastrointestinal tract.

Angiography should be reserved for patients with life-threatening bleeding that are not surgical candidate or in whom localization or embolization of lesions is needed prior to surgical resection [5].

So, endoscopic, surgical, or radiological treatment can be considered in these situations. In this particular case the option was surgical because the appearance of the lesion on capsule enteroscopy resembled a GIST. Unexpectedly, the observed lesion was an angiodysplasia that resembled a tumor given its protruded appearance as a polypoid lesion, in opposition to the regular aspect of a red flat lesion that is usually described on the literature.

This case demonstrates a different endoscopic presentation of an angiodysplasia responsible for massive mild-GI bleeding. The new enteroscopy methods became essential in these cases but there is still a place for IOE.
